# A Practical Handbook of Medical Chemistry

**Published:** 1850-10

**Authors:** 


*A Practical Handbook of Medical Chemistry.* By JOHN E. BOWMAN, Fellow of the Chemical Society, Demonstrator of Chemistry in King's College, London, and Author of "Practical Chemistry." Philadelphia: Lea & Blanchard, 1850.

The little work whose title heads this notice fills the vacancy that seemed to be left in the "Introduction to Practical Chemistry" by the same author, which was brought to the notice of our readers about a year ago. To the student of medicine and the practitioner this will prove particularly acceptable, by supplying him, in a clear and compendious form, with information too rarely obtained from his teachers, especially in relation to the analysis of urine, blood and the detection of poisons in organic mixtures, and which most frequently he obtains only through the assistance of others, who have more leisure or more experience than himself. The work is divided into five parts: Part I. is of URINE. The first Chapter contains an account of healthy urine, and its various constituents, Chapter 2. The Qualitative Analysis of Healthy urine. Chapter 3. Average composition of Healthy urine. Chapter 4. Morbid urine, and the various extraneous matters met with in it. Chapter 5, is on the Qualitative examination of urine suspected to contain either an unnatural proportion of some one or more of the usual ingredients, or some abnormal matter. The various abnormal substances are detailed, and plain directions given for their detection. Chapter 6, is on the Examination of Morbid urine, the nature of which is unknown. Chapters 7 and 8, relate to the qualitative analysis of diabetic and albuminous urine. Part II. relates to Calculi and their concretions, both urinary and biliary. Part III. is on the blood—its quantitative and qualitative analysis. Part IV. describes Milk, Mucus Pus, Bone, etc.; and Part V. and last treats of the detection of poisons in organic mixtures, etc.' of the former of which the most common are detailed, in connection with their tests; to which is added the method of examining an organic mixture suspected to contain some mineral poison, the nature of which is unknown. From this list of the contents of the work, our readers will be enabled to form an estimate of the amount of useful matter contained in it. We commend it most cordially to their sedulous examination as a reliable handbook, more especially to those whose locations do not admit of ready appeal to others more conversant with these important aids to diagnosis.

